# Hydrogen Purification from Compact Palladium Membrane Module Using a Low Temperature Diffusion Bonding Technology

**DOI:** 10.3390/membranes10110338

**Published:** 2020-11-12

**Authors:** Duck-Kyu Oh, Kwan-Young Lee, Jong-Soo Park

**Affiliations:** 1 Department of Chemical and Biological Engineering, Korea University, 145 Anam-ro, Seoul 02841, Korea; ohdk@kier.re.kr (D.-K.O.); kylee@korea.ac.kr (K.-Y.L.); 2 Energy Conversion & Storage Materials Laboratory, Korea Institute of Energy Research, 152 Gajeong-ro, Daejeon 34129, Korea

**Keywords:** hydrogen, carbon dioxide, purification, palladium membrane, compact module, diffusion bonding

## Abstract

This study investigates a compact palladium membrane module (CPMM) for hydrogen purification, assembled by diffusion bonding at a low-temperature (450 °C). This CPMM resulted in hydrogen (H_2_) flux of 18.3 mL cm^−2^ min^−1^ with H_2_/N_2_ selectivity of over 1100. The H_2_ purification test using a 60% H_2_/40% CO_2_ mixed gas confirmed that the CPMM can separate H_2_ with a concentration of more than 99%, with a pressure difference of 5 bar. Moreover, the volume of the diffusion bonded membrane module is decreased by 81.4% than the flame-type membrane module pre-studied in our laboratory.

## 1. Introduction

High-purity H_2_ gas is required for many chemical, industrial, and electronic applications such as ammonia production, steel and glass manufacture, and electrical generator cooling, respectively. Since it is nontoxic and an efficient fuel source, H_2_ is considered a clean energy carrier [1,2]. Fuel cell systems also require high-purity H_2_ for portable power. Consequently, the demand for high-purity H_2_ is increasing [3,4].

Hydrogen can be produced from fossil fuels by thermochemical processes like steam reforming of natural gas and by various water-splitting methods, e.g., thermochemical, photo-electro-chemical, photo-biological, and electrolysis. Over 95% of H_2_ gas is produced from fossil fuels [5]. Hydrogen production from fossil fuels typically comprises four steps: Desulfurization, high-temperature shift reaction, low-temperature shift reaction, and H_2_ purification [6,7]. The produced hydrogen can be purified via pressure swing adsorption (PSA), cryogenic distillation, and membrane separation. Among these, the PSA and cryogenic distillation methods are commercially available [8,9]. However, they are both intensive in terms of technical expertise, energy, and infrastructure requirements during operation [10,11]. The membrane separation technique has relatively low energy and cost requirements during operation. Furthermore, palladium membranes are excellent for H_2_ separation and purification because of their high permeability and high selectivity for H_2_ [11,12,13]. The hydrogen separation mechanism of the palladium membrane is basically depending on the hydrogen partial pressure. The Hydrogen molecules were dissociated into hydrogen atoms at the surface of the palladium membrane, and then diffusion into the palladium membrane. The separated hydrogen atoms through the membrane were recombined with hydrogen molecules on the opposite surface [14].

When using palladium based dense membranes, the design and configuration of the membrane module are critical for successful application in H_2_ separation and purification. In particular, the configuration of compact modules is necessary in small-scale H_2_ purification. Membrane modules can be divided into the several types depending on the membrane shape, e.g., flange [15,16,17] and tubular [11,13,15,18,19] modules. Though it may be easy to scale-up these membrane modules, ensuring compact module configuration poses difficulty.

Micro channel reactors (MCRs) are advantageous in terms of their considerable heat and mass transfer, large surface-area-to-volume ratio, compactness, and easy integration [20]. This paper reports for the first time the application of an MCR in configuring a compact and multi-stackable membrane module. For this purpose, it is vital to examine the methods for effectively assembling stainless steel (SS) components to prepare the MCR module. Typically, diffusion bonding was performed at a high-temperature (>1000 °C) using 316L SS in order to obtain a compact palladium membrane module (CPMM) [21]. However, because of the exposure of the CPMM to the harsh conditions of high-temperature and high vacuum, defects can be generated within the palladium membrane during its preparation. Therefore, the plate surfaces were modified such that the diffusion bonding could be performed at a low-temperature (450 °C) to ensure minimum damage to the palladium membrane. The module performance test was performed to determine the feasibility of the CPMM. The above-mentioned H_2_ separation and purification modules has disadvantages in terms of scale-up and compactness due to their complicated structure and design. accordingly, we designed an MCR type H_2_ separation and purification module to provide easy assembly and compact with diffusion bonding technology.

## 2. Materials and Method

### 2.1. Design and Manufacture of the CPMM

**Figure 1:**
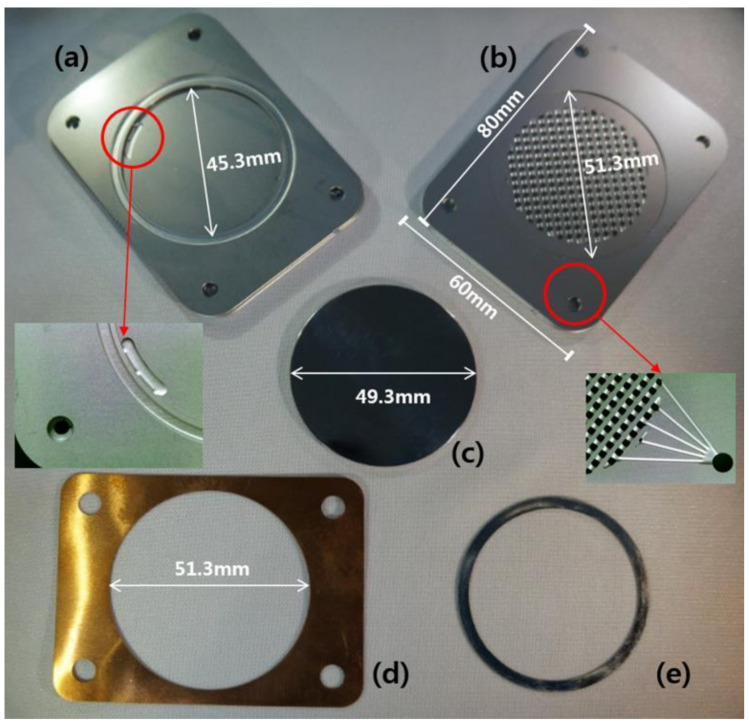
Constituent photographs of compact palladium membrane module (CPMM); etched 316L stainless steel (SS) plates of (**a**) feed stream and (**b**) permeate stream, (**c**) disc-shaped Pd-based membrane, (**d**) Cu foil, (**e**) Ag gasket.

**Figure 2:**
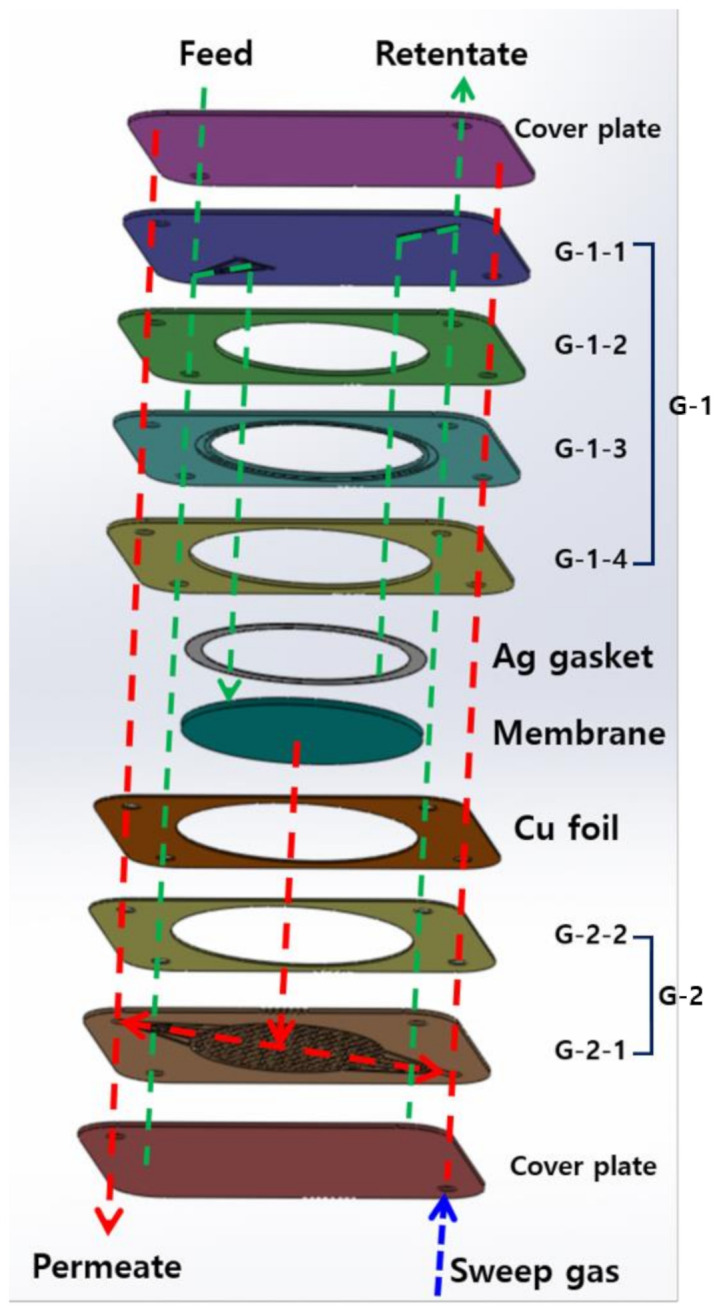
The components and gas path in the CPMM; Green line: Feed stream gas path, Red line: Permeate stream gas path.

The palladium based membranes were manufactured by our laboratory using the preparation procedures explained in a previous work [22,23]. Palladium was deposited on the prepared Porous nickel support (PNS) (49.3 mm) at 450 °C, 20 mTorr under Ar using a DC/RF magnetron sputter (Korea Vacuum Tech Co., Ltd., Daegu, Korea). Then, polishing and heat treatment were performed to produce a dense palladium membrane with improve selectivity. Figure S1 shows the cross-section SEM image of palladium membrane. The estimate thickness of palladium membrane layer was 2.7 μm. The constituents of the CPMM are shown in Figure 1. The module is composed of etched 316L SS plates (a, b), a palladium membrane (c), a Cu foil (d), and a Ag gasket (e). The 316L SS plates were chemically etched with various channels and holes. Figure 2 shows the components and gas path (retentate stream (G-1) and a permeate stream (G-2)) in the CPMM. The designed 316L SS plates consist of a cover plate (two sheets) of 1 mm thickness, a feed gas distribution plate (G-1-1), a plate for protection of the knife-edge (G-1-2), a knife-edge plate for membrane sealing (G-1-3), a membrane holder plate (G-1-4 and G-2-2), and permeate gas collection plates (G-2-1). Three-dimensional (3-D) porous channels were formed on the G-2-1 plate to sufficiently distribute the permeate gas through the Palladium membrane. 

The G-1 and G-2 groups were modified using the following method to decrease the diffusion bonding temperature and to easily assemble components.

(1) Each G-1 and G-2 part was diffusion bonded using hot-press (Samyang Ceratech Co., Ltd., Incheon, Korea) under high vacuum (3.0 × 10^−6^ Torr) and 900 °C
(2) The surfaces of bonded G-1 and G-2 (G-1-4 in G-1 and G-2-2 in G-2) were peened using a peening machine (IEPCO PEENMATIC 550, Swiss Instruments Limited Co., Ltd., Mississauga, Canada) to increase the surface roughness using 316L SS powder by spraying method.
(3) Ni and Cu were deposited over the peened surfaces of the G-1 and G-2 using the DC magnetron sputtering method.
(4) G-1 and G-2 with Ni and Cu deposition were thermally treated under H_2_ condition at 700 °C for 2 h in the muffle furnace.

We analyzed the morphology, average surface roughness, and surface area of the modified surfaces using a color confocal microscope (H1200, Lasertec Co., Ltd., Hwa-seong, Korea). The CPMM was completed by diffusion bonding with modified G-1 and G-2, Cu foil (thickness 0.4 mm), Ag gasket (thickness 0.25 mm), and Palladium membrane. The diffusion bonding was performed at 450 °C for 5 h under high vacuum (3.0 × 10^−6^ Torr) and 20 Ton in the hot press. The leak test of the CPMM was performed at a pressure difference of 5 bar at room temperature.

### 2.2. Permeation Test Method of Hydrogen

**Figure 3:**
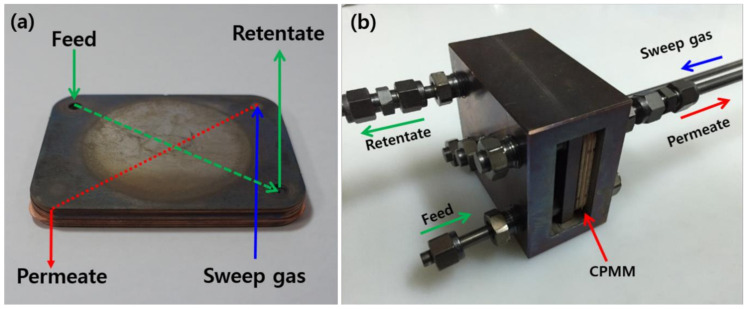
The photograph of (**a**) diffusion bonded CPMM and (**b**) the assembled H_2_ permeation apparatus.

The photograph of the diffusion bonded CPMM is shown in Figure 3a. Feed gas was introduced to the membrane through the G-1 group. Then, the permeated gas through the membrane was an exhaust-to-permeation stream through the G-2. The photograph of H_2_ permeation apparatus for the permeation experiment was shown in Figure 3b. The diffusion bonded CPMM was fixed and connected with a SS tube. The CPMM was heated in an electric furnace with a programmable temperature controller. The temperature of the furnace was controlled by a k-type thermocouple placed close to the membrane surface in the retentate stream. To prevent phase transition of the palladium, N_2_ was supplied up to 300 °C [24]. The permeation rates of H_2_ and N_2_ as a single gas were measured by a digital soap bubble flow meter (Gilibrator, SENSIDYNE Co., Ltd., Petersburg, FL, USA) at a pressure difference of 1 to 20 bar and 400 °C.

We performed the H_2_ separation tests using the H_2_/CO_2_ mixture gas at a pressure difference of 1–20 bar and a temperature of 400 °C with a feed gas flow rate range of 1.0–1.5 L min^−1^. We analyzed the H_2_ concentration of the permeate stream by Gas Chromatography. (G.C., 7890A, Agilent technology Co., Ltd., Santa Clara, CA, USA) equipped with a carboxen 1010 PLOT capillary column and thermal conductivity detector (TCD). Upon completion of the permeation testing, the membrane module was slowly cooled under N_2_. For cross-sectional analysis of the low-temperature diffusion bonded module, we used water-jet cutting and characterized the cut module by field emission scanning electron microscopy (FE-SEM, HITACHIS-4700). 

## 3. Result and Discussion

### 3.1. Characterization of Membrane and CPMM

**Figure 4:**
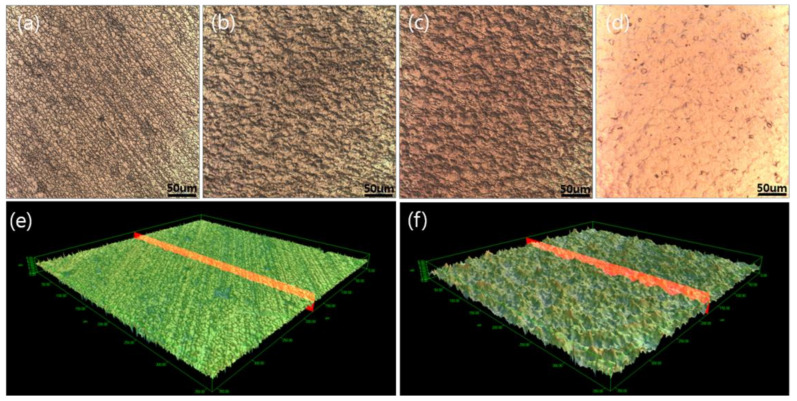
The surface image of modified 316L SS plate by color confocal microscope; 2-D image: (**a**) Fresh, (**b**) peening, (**c**) Ni-Cu deposition, (**d**) thermal treatment; 3-D image: (**e**) Fresh and (**f**) peening.

The average surface roughness (Ra) and surface area of the pretreated G-1 and G-2 groups were analyzed using a color confocal microscope, and the results are presented in Table 1. As shown in Table 1, The Ra values and areas of the surfaces modified by peening, Ni-Cu deposition, and thermal treatment were significantly different from those of the fresh, non-treated surfaces of G-1 and G-2. The Ra value of the peened surface was significantly higher (0.446 μm) than that of the fresh surface (0.167 μm). The increases in surface roughness and surface area of G-1 and G-2 were directly associated with effective diffusion bonding. This association means that the components can be effectively diffusion bonding at the low-temperatures. As shown in Figure 4, the surface morphologies of G-1 and G-2 obtained at different stages of surface modification were analyzed using a color confocal microscope. Figure 4a–d shows two-dimensional (2-D) surface images of the fresh and modified surfaces. After the peening process, the surface morphology showed higher surface roughness and surface area than those of the fresh surface morphology. Conversely, the surface morphology showed decreased surface roughness compared to the Ni-Cu-deposited surface by sputtering. Figure 4e,f shows the 3-D morphology of the untreated and peened surfaces. After the peening process, many pin-points were observed on the surface compared to the untreated surface of plate. The generated pin-points, designated with red color, were subjected locally to very high pressure during the diffusion-bonding step, resulting in easy assembly between the G-1 and G-2 groups at low-temperatures.

**Figure 5:**
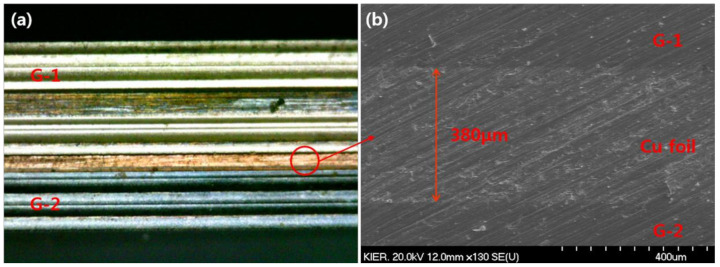
The cross-sectional image of diffusion bonded CPMM; (**a**) photograph, (**b**) SEM image of diffusion bonded layer.

Figure 5 shows the cross-sectional image of the CPMM. As shown in Figure 5a, the height of assembled module was obtained 7.9 mm. Figure 5b shows the low-temperature bonded layers between the G-1 and Cu foil and the G-2 and Cu foil can be seen to be defect free. 

### 3.2. Hydrogen Permeation Test of the CPMM

The leak test of the CPMM installed with the palladium membrane was carried out using the N_2_ at room temperature. N_2_ of the permeate stream was not detected at a pressure difference of 1 bar and at room temperature. After confirming the leak-tightness of the CPMM, we carried out various performance tests. The performance of the membrane may be expressed in terms of a flux and selectivity as defined by Equations (1) and (2), respectively:

Flux = *F*/*A* (1)

H_2_ Selectivity = H_2_ flow in permeate stream/N_2_ flow in permeate stream (2)

where *F* represents the H_2_ flow through the membrane in the permeate stream and *A* is the effective area of the membrane (cm^2^).

**Figure 6:**
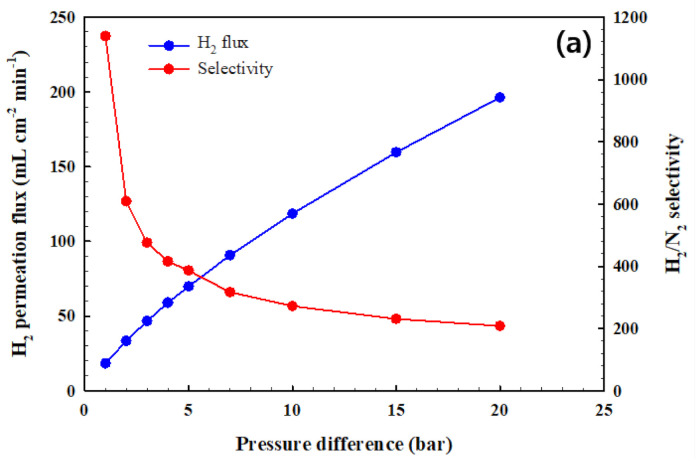

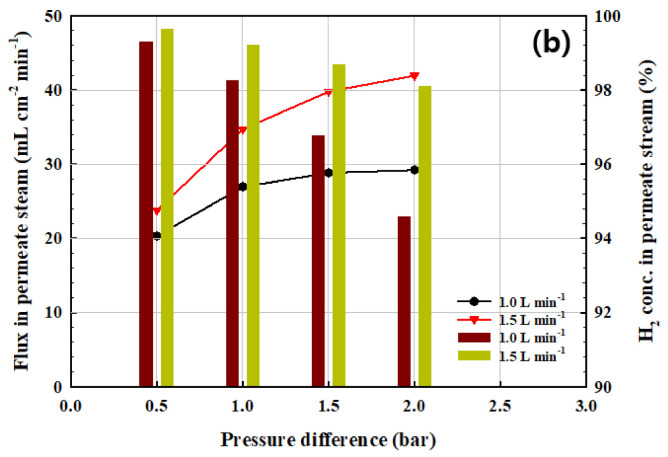
The performance test of CPMM as a function of pressure difference at the temperature of 400 °C; (**a**) the hydrogen permeation flux and H_2_/N_2_ selectivity, (**b**) the flux and H_2_ concentration of permeation gas using the H_2_/CO_2_ mixture gas; vertical bar plot: H_2_ concentration, line plot: H_2_ flux.

We fabricated CPMM by diffusion bonding carried out a long-term stability test at a pressure difference of 1bar and 400 °C. As shown in Figure S2, the H_2_ permeation and H_2_ selectivity remained constant for ~365 h. Figure 6 shows the H_2_ permeation flux and selectivity using the pure gas and the flux and concentration of H_2_ in the permeation using the H_2_/CO_2_ mixture gas as a function of pressure difference at 400 °C. As shown Figure 6a, The H_2_ permeation flux significantly increased with pressure difference. The driving force for H_2_ separation is the pressure difference between the retentate and permeate. The flux of H_2_ through the CPMM ranged from 18.3 to 196.3 mL cm^−2^ min^−1^, depending on the pressure difference (1–20 bar). However, the H_2_/N_2_ selectivity significantly decreased from 1138 to 208 as the pressure difference was increased from 1 to 20 bar. The decrease in H_2_/N_2_ selectivity is considered to be due to the generated palladium fracture and defect of the membrane surface during the diffusion bonding of the CPMM. As shown in Figure 6b, the H_2_ separation test was performed using the 60% H_2_/40% CO_2_ mixture gas. The H_2_ separation capacity of the CPMM was examined with the H_2_/CO_2_ mixture gas flow rate of 1.0–1.5 L min^−1^ at 400 °C and a pressure difference ranging from 5 to 20 bar. The H_2_ flux increases with increasing pressure in the retentate stream. However, the H_2_ concentration decreases with increasing pressure due to H_2_/N_2_ selectivity, as shown in Figure 6a. The H_2_ concentration was >99% and H_2_ flux was 34.7 mL cm^−2^ min^−1^ in the permeate stream when the feed rate was 1.5 L min^−1^ and the pressure difference was 10 bar.

**Figure 7:**
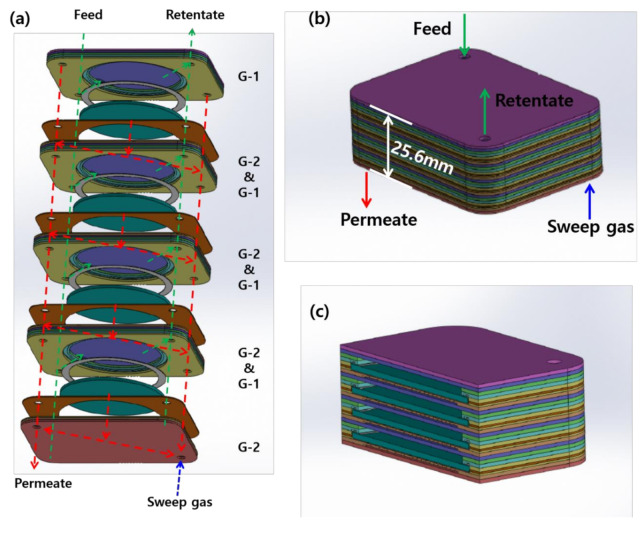
The schematic drawing of multi stacked CPMM; (**a**) layout of the gas path in the CPMM, (**b**) multi stacked CPMM, (**c**) cross-section of multi stacked CPMM.

Figure 7 shows the drawing of multi stacked CPMM. The capacity of the multi stacked CPMM can be increased by scaling up the unit modules, as shown in Figure 7. The H_2_ flow of 2.1 L min^−1^ with H_2_ concentration of >99% will be produced by the multi-stacking membrane module with four membranes. Moreover, the module height is 25.6 mm and the module volume is 122.8 cm^3^. The module volume was 553.4 cm^3^ in the case of the frame-type membrane module with four membranes pre-studied in our laboratory [17]. The volume of multi stacked CPMM will be able to decrease by 77.8% at using the four membranes, compared with the frame-type membrane module.

**Figure 8:**
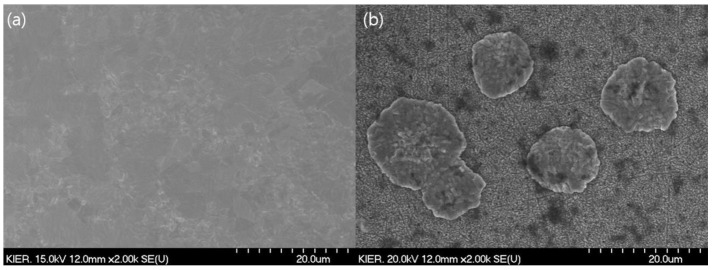
The surface SEM image of Palladium membrane; (**a**) fresh membrane, (**b**) membrane after the diffusion bonding and permeation test.

Figure 8 shows the surface SEM images of the fresh and used palladium membranes in the CPMM. As shown Figure 8a, The surface of the fresh membrane was clear. However, despite diffusion bonding at a relatively low-temperature of 450 °C, some palladium fracture were partially formed on the surface of the palladium membrane, as seen in Figure 8b. This result indicates that the membrane morphology is affected and some sintering occurs on the top layer of the palladium membrane even when diffusion bonding is carried out at a low-temperature of 450 °C under high-vacuum conditions.

## 4. Conclusions

The CPMM was prepared and constructed for H_2_ separation. The G-1 and G-2 groups were prepared after the high-temperature diffusion bonding to reduce damage to the membrane. Next, the G-1 and G-2 groups was modified for low-temperature diffusion bonding of the module. The effective diffusion bonding area of the surface of the 316L SS plate was increased using peening, Ni-Cu deposition, and thermal treatment, resulting in perfect diffusion bonding of the membrane module at low-temperatures. The diffusion bonded module was obtained by height 7.9 mm, and volume 37.9 cm^3^. In addition, the volume of the diffusion bonded module than the pre-studied frame-type module were decreased by 81.4% [23]. Even though diffusion bonding was entirely performed at a low-temperature of 450 °C, a Palladium fracture was generated due to high-vacuum conditions on the membrane surface. The H_2_ permeation flux and the H_2_/N_2_ selectivity tests were conducted at a pressure difference of 1 bar, and a H_2_ flux of 18.3 mL cm^−2^ min^−1^ and H_2_/N_2_ selectivity of 1138 at 400 °C were observed in the CPMM. In the hydrogen separation test of H_2_/CO_2_ mixture gas, the H_2_ flux was 34.7 mL cm^−2^ min^−1^ and the H_2_ concentration was confirmed to be over 99.0%. However, the H_2_/N_2_ selectivity of the membrane decreased with increasing pressure difference. In the present study, we suggest that the CPMM can be used for compact H_2_ purifier. Further research is needed on the assembly methods to minimize membrane damage.

## Figures and Tables

**Table 1:** The surface properties of modified commissure by color confocal microscope.

Process	Ra (µm)	Surface Area (mm^2^)
Fresh	0.167	0.128
Peening	0.446	0.132
Ni-Cu deposition	0.582	0.133
Thermal treatment	0.520	0.132

## Supplementary Materials

The following are available online at https://www.mdpi.com/2077-0375/10/11/338/s1, Figure S1: Long-term stability test of CPMM; Temperature was 400℃; Pressure difference was 1 bar, Figure S2: Cross-sectional image of the prepared membrane; (a) FE-SEM, (b) FE-SEM/EDS.
