# Supplementary material for: Hydrogen Purification from Compact Palladium Membrane Module Using a Low Temperature Diffusion Bonding Technology
Source: Membranes (Basel). 2020 Nov 12;10(11):338. doi: 10.3390/membranes10110338 (PMC7696550; doi:10.3390/membranes10110338)
Supplement: Supplementary file 1 [file membranes-10-00338-s001.pdf]

membranes

Supplementary materials

Hydrogen purification from compact palladium membrane module using a low temperature diffusion bonding technology

Duck-Kyu Oh [1,2], Kwan-Young Lee [1] and Jong-Soo Park [2,*]

[1] Department of Chemical and Biological Engineering, Korea University, 145 Anam-ro, Seoul 02841, Korea; ohdk@kier.re.kr (D.-K.O.); kylee@korea.ac.kr (K.-Y.L.)
[2] Energy Conversion & Storage Materials Laboratory, Korea Institute of Energy Research, 152 Gajeong-ro, Daejeon 34129, Korea
* Correspondence: deodor@kier.re.kr; Tel.: +82-42-860-3667

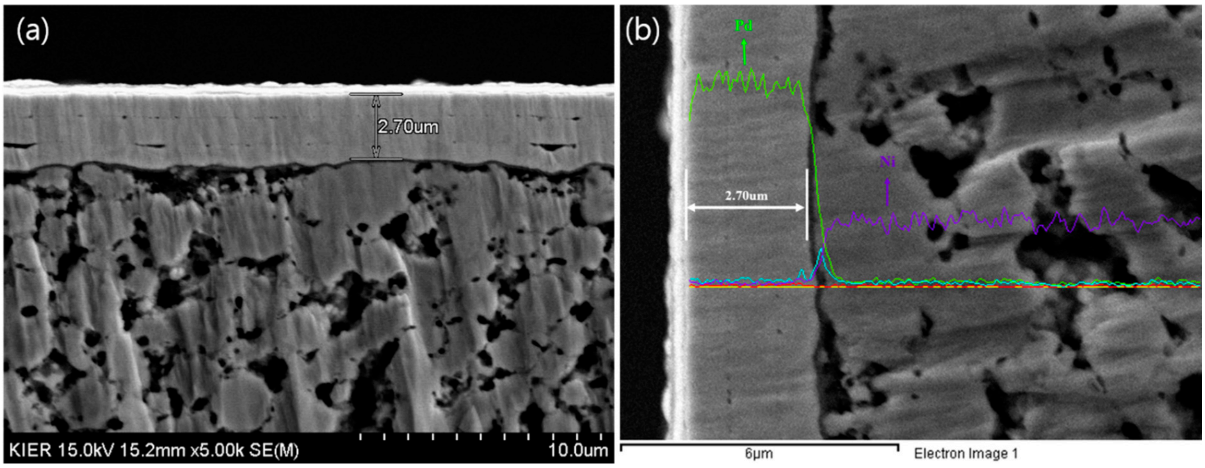

Figure S1. Cross-sectional image of the prepared Pd membrane.; (a) FE-SEM, (b) FE-SEM/ EDX

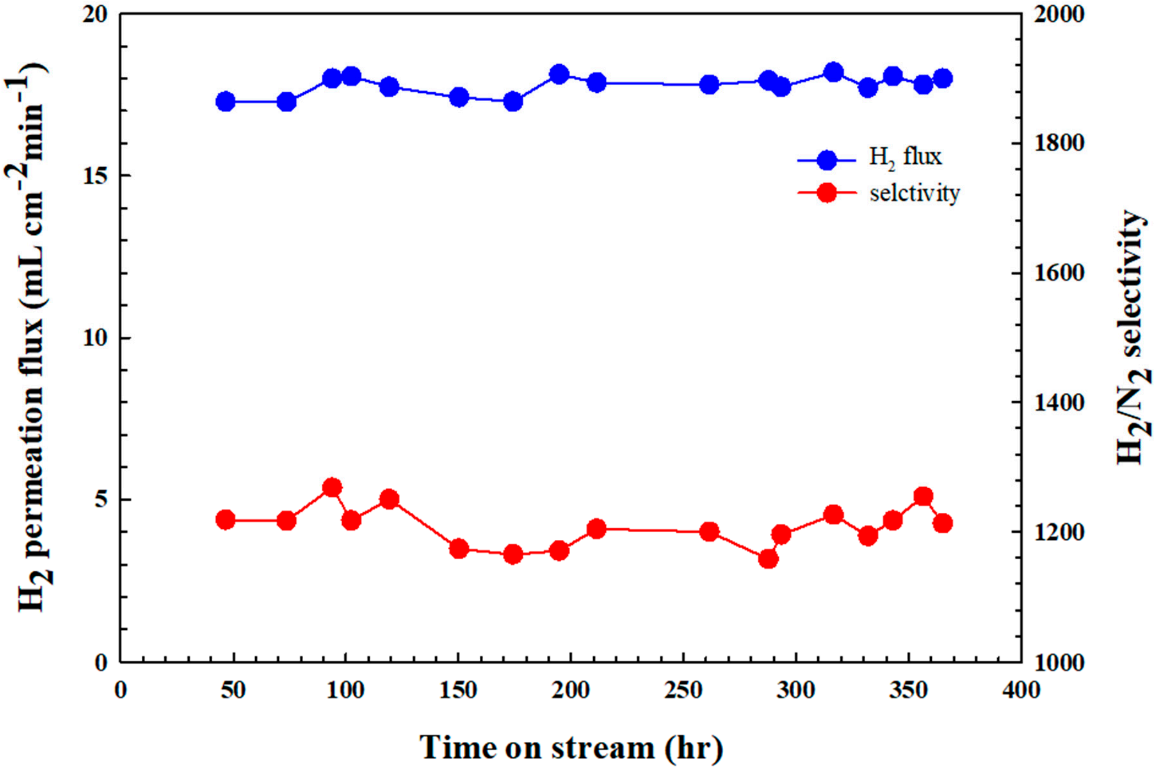

Figure S2. Long-term stability test of CPMM; Temperature was 400 °C; Pressure difference was 1 bar.
